# Supplementary material for: STOP Strategy to Inhibit P. falciparum and S. aureus Growth: Molecular Mechanism Studies on Purposely Designed Hybrids
Source: Antibiotics (Basel). 2025 Oct 3;14(10):991. doi: 10.3390/antibiotics14100991 (PMC12561106; doi:10.3390/antibiotics14100991)
Supplement: Supplementary file 1 [file antibiotics-14-00991-s001.zip › antibiotics-3869155-supplementary.pdf]

Supporting information

STOP strategy to inhibit *P. falciparum* and *S. aureus* growth: molecular mechanism studies on purposely designed hybrids

Beatrice Gianibbi [1], Riccardo Corina [2,§], Nicoletta Basilico [3], Ottavia Spiga [1], Silvia Gobbi [2], Federica Belluti [2], Giovanna Angela Gentilomi [4,5], Silvia Parapini [6], Francesca Bonvicini [4,*] and Alessandra Bisi [2,*]

[1] Department of Biotechnology, Chemistry and Pharmacy, University of Siena, Via Aldo Moro 2, 53100, Siena, Italy
[2] Department of Pharmacy and Biotechnology, Alma Mater Studiorum-University of Bologna, Via Belmeloro 6, 40126 Bologna, Italy
[3] Department of Biomedical, Surgical and Dental Sciences, Università degli Studi di Milano, Via Carlo Pascal, 36, Milano 20133, Italy
[4] Department of Pharmacy and Biotechnology, Alma Mater Studiorum-University of Bologna, Via Massarenti 9, 40138, Bologna, Italy
[5] Microbiology Unit, IRCCS Azienda Ospedaliero-Universitaria di Bologna, Via Massarenti 9, 40138 Bologna, Italy
[6] Department of Biomedical Sciences for Health, Università degli Studi di Milano, Via Carlo Pascal, 36, Milano 20133, Italy

§ Present address: CIRAD, UMR Qualisud-Avenue Agropolis 34398-Montpellier Cedex 5-France
* Correspondence: francesca.bonvicini4@unibo.it; alessandra.bisi@unibo.it

Table S1. MIC values obtained for the PTZ-quinoline hybrid compounds measured for the reference microbial strains

	MIC (µM)		
**Compound**	***S. aureus* ATCC 25923**	***E. coli* ATCC 25923**	***C. albicans* ATCC 10231**
**1**	>100	>100	>100
**2**	>100	>100	>100
**3**	>100	>100	>100
**4a**	12.5	>100	>100
**4b**	6.25-12.5	>100	>100
**5a**	12.5	>100	>100
**5b**	6.25-12.5	>100	>100
**6a**	>100	>100	>100
**6b**	50	>100	>100
**7**	>100	>100	>100
**8**	>100	>100	>100
**GEN***	6.6	1.03	n.d
**AMP***	4.2	67.3	n.d.
**FLC***	n.d.^	n.d.	0.82

*GEN: Gentamicin; AMP: Ampicillin; FLC: Fluconazole.

^n.d. not detemined

Table S2. Antibiotic-resistance profile of the clinical isolates of *S. aureus*

Clinical isolate	**Antibiotic-resistance profile**
**MRSA 1**§	GEN^{S}, LVX^{R}, $\mathbf{OX^{R}}$, $\mathbf{P^{R}}$, TE^{S}, TEC^{S}, SXT^{S}, VA^{S}
**MRSA 2**§	GEN^{S}, LVX^{R}, $\mathbf{OX^{R}}$, $\mathbf{P^{R}}$, TE^{S}, TEC^{S}, SXT^{S}, VA^{S}
**MRSA 3**§	$\mathbf{CM^{R}}$, $\mathbf{E^{R}}$, GEN^{S}, $\mathbf{LVX^{R}}$, $\mathbf{OX^{R}}$, $\mathbf{P^{R}}$, TEC^{S}, TE^{S}, SXT^{S}, VA^{S}
**MRSA 4**§	CM^{S}, E^{S}, GEN^{S}, $\mathbf{LVX^{R}}$, $\mathbf{OX^{R}}$, $\mathbf{P^{R}}$, TE^{S}, TEC^{S}, SXT^{S}, VA^{S}
**MRSA 5**§	CM^{S}, E^{S}, GEN^{S}, $\mathbf{LVX^{R}}$, $\mathbf{OX^{R}}$, $\mathbf{P^{R}}$, TE^{S}, TEC^{S}, SXT^{S}, VA^{S}

CM = Clindamicyn; E = Erythromycin; GEN = Gentamicin; P = Penicillin; LVX = Levofloxacin; OX = Oxacillin; TE = Tetracycline; TEC = Teicoplanin; SXT = Trimethoprim/Sulfamethoxazole; VA = Vancomycin

R = Resistant; S = Susceptible; I = Intermediate, as defined following the EUCAST guidelines

§*Staphylococcus* species resistant to oxacillin were declared, by convention, methicillin-resistant.

Table S3. IC_{50} ranges (µM) obtained for MRSA and reference strains

S. aureus	**4a**	**4b**	**5a**	**5b**
**ATCC 25923**	7.34 – 7.64	3.97 – 5.75	5.41 – 8.05	3.31 – 6.75
**MRSA (n = 5)**	7.75 – 8.98	4.42 – 6.48	6.40 – 9.63	6.18 – 10.34

Table S4. Molecular docking scores of compounds 1-8 within the evaluated binding sites of *S. aureus* NDH-2 protein.

Compound	Docking score (kcal/mol) FAD binding site	Docking score (kcal/mol) NAD^+/NADH binding site	Docking score (kcal/mol) Q binding site
**1**	-8.159	-8.153	-8.537
**2**	-8.353	-8.007	-7.837
**3**	-8.551	-8.120	-7.211
**4a**	-8.757	-7.158	-9.794
**4b**	-8.281	-7.306	-8.129
**5a**	-9.059	-7.583	-9.575
**5b**	-9.297	-7.763	-8.628
**6a**	-9.198	-8.174	-9.732
**6b**	-9.856	-8.312	-11.174
**7**	-9.718	-8.011	-8.325
**8**	-9.895	-8.070	-9.590
**NAD^+***	-	-6.420	-
**HQNO***	-	-	-6.170
**FAD***	-17.036	-	-

*NAD^+, HQNO and FAD are reported as reference scoring compounds.

Table S5. Molecular docking scores of compounds 1-8 within the evaluated binding sites of *P. falciparum* NDH-2 protein.

Compound	Docking score (kcal/mol) FAD binding site	Docking score (kcal/mol) NAD^+/NADH binding site	Docking score (kcal/mol) Q binding site
**1**	-6.446	-7.721	-7.983
**2**	-6.937	-6.455	-7.931
**3**	-6.662	-8.374	-8.014
**4a**	-7.150	-8.150	-8.206
**4b**	-7.061	-8.274	-10.208
**5a**	-7.223	-8.136	-8.911
**5b**	-7.435	-8.505	-9.374
**6a**	-7.604	-8.438	-7.987
**6b**	-7.882	-8.600	-7.566
**7**	-7.970	-7.274	-8.747
**8**	-7.867	-8.446	-9.120
**NAD^+***	-	-8.879	-
**HQNO***	-	-	-7.799
**FAD***	-14.378	-	-

*NAD^+, HQNO and FAD are reported as reference scoring compounds.

Figure S1. Dose-response curves of the active PTZ-quinoline hybrid compounds against *S. aureus* ATCC 25923

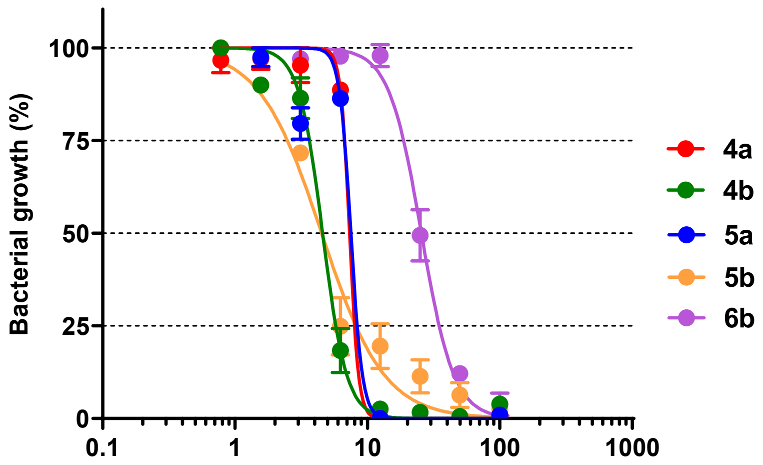

Symboles represent mean values with standard deviations, and lines define the curves obtained from nonlinear regression analysis (GraphPad Prism version 9.4.1). Percentage values are relative to the positive control (bacteria grown in regular medium).

Figure S2. IC_{50} values measured for *S. aureus* ATCC 25923

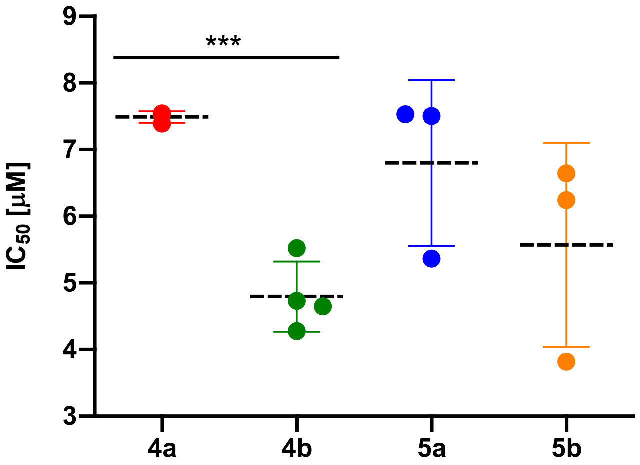

Symboles represent IC_{50} values obtained in different experiments against *S. aureus*, and the dotted lines define the mean values for the PTZ-quinoline hybrid compounds. A statistically difference is measured comparing the triazole derivatives **4a** and **4b** (***$p<0.0001$ unpaired t test).

Figure S3. Hemolytic activity, expressed in percentage values, for the compounds measured on hRBCs.

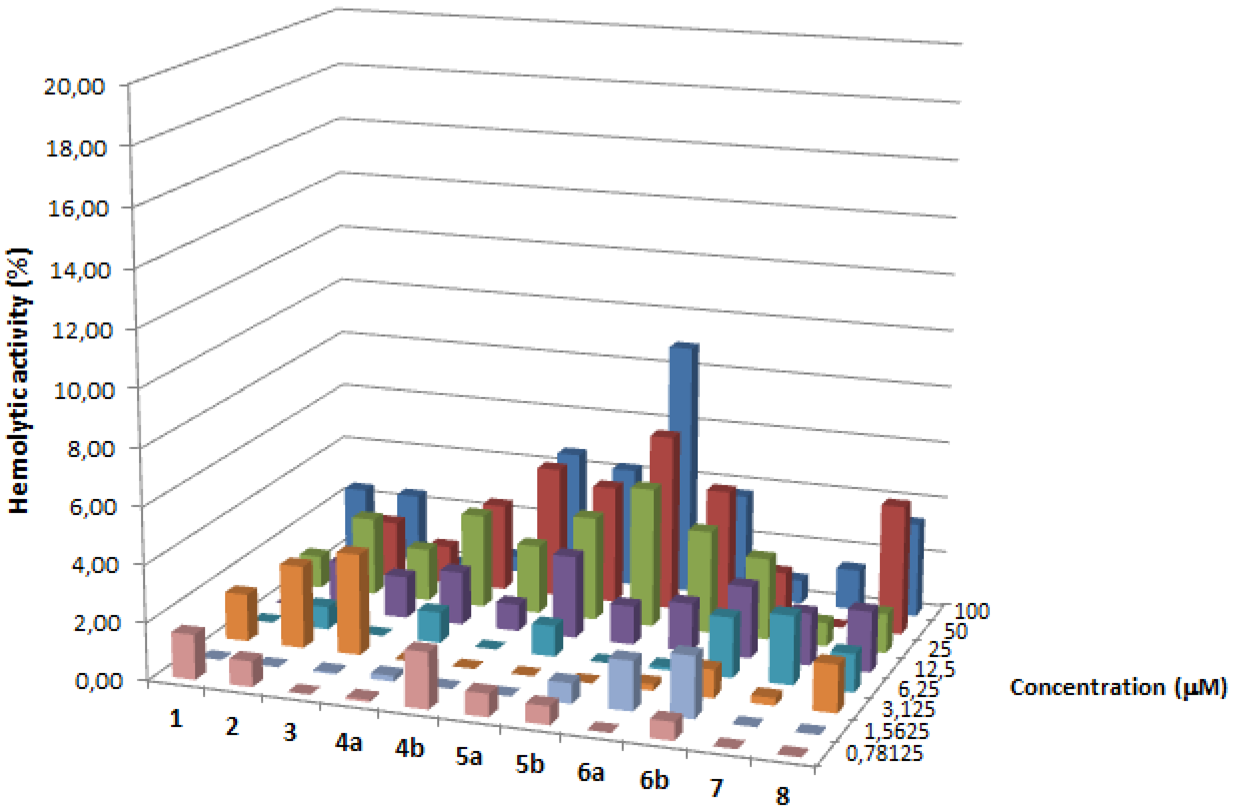

Figure S4. Sequence alignment between *S. aureus* and *P. falciparum* NDH-2 protein isoforms.

```
CLUSTAL O(1.2.4) multiple sequence alignment


s.aureus        -----------------------------------MAQDRKKVLVLGAGYAGLQTVTKLQ  25
p.falciparum    MLVKFRKCGQANIFRSISNVRKIYNVAKNNLKNNKDIERKEKIIILGSGWGGFNFLLNID  60
                                                     : ::*:::**:*:.*:: : :::

s.aureus        KAISTEEAEITLINKNEYHYEATWLHEASAGTLNYEDVLYPVESVLKKDK---VNFVQAE  82
p.falciparum    ----FKKYDVTLISPRNYFTFTPLLPCLCSGTLSVNVCTESIRNFLRKKNGYCGNYLQLE  116
                     :: ::***. .:*.  :  *   .:***. :     :...*:*.:    *::* *

s.aureus        VTKIDR-----DAKKVETNQGIYDFDILVVALGFVSETFGIEGMKDHAFQIENVITAREL  137
p.falciparum    CTDVFYEDKYINCIDIENNKVKLFYDYLIIAVGAKTNTFNINGVDKYAYFVKDIDDALKI  176
                 *.:       :. .:*.*:    :* *::*:*  ::**.*:*:..:*: ::::  * ::

s.aureus        SRHIEDKFA---NYAASKEKDDNDLSILVGGAGFTGVEFLGELTDRIPEL-CSKYGVDQN  193
p.falciparum    RKKFLDILEKCTLPNISNEEKKKMLHVAVVGGGPTGVEVTAEFADFINKEVKINYKDIFN  236
                 ::: * :        *:*:..: * : * *.* ****. .*::* * :    :*    *

s.aureus        KVKITCVEAAPKMLPMFSEELVNHAVSYLEDRGVEFKIATPIVACNEKGFVVE---VDGE  250
p.falciparum    FISISIIEGGNNLLPTFTQNISDFTKENFHNLNINVLTNYYVIDVDKHSFHIQSSLNKNE  296
                 :.*: :*.. ::** *:::: :.: . :.: .::.     ::  :::.* ::    ..*

s.aureus        KQQLNAGTSVWAAGVRGSKLMEESFEGVKRGR----IVTKQDLT--INGYDNIFVIGDCS  304
p.falciparum    KKKLSYGLLIWASGLAQTTLIQKFLKTIPVQANNAILKVDEKLRVIGIPSNNIYAIGDCK  356
                *::*. *  :**:*:  :.*::: :: :        : ..:.*       :**:.****.

s.aureus        AFIPAGEERPLPTTAQIAMQQGESVAKNIKRILNGEST--------EEFEYV--DRGTVC  354
p.falciparum    KIQPKLLHEHTNEI--IKILTGNKLTSEALKLKQSELTKTFPQLSISKWDYEKNKKGEMT  414
                 : *   ..       * :  *:.::.:  :: :.* *        .:::*   .:* :

s.aureus        SLGSHDGV---GMVFGKPIAG-----KKAAFMK----KVIDTRAVFKIGGIGL-------  395
p.falciparum    PQQFHDYLFEIDKNYKSPTPTAQNAKQEAYYLSNVFNNFIHTNQKFNIPSFIEKWKGSLA  474
                    ** :   .  : .*        ::* ::.    :.*.*.  *:* .:

s.aureus        --------------AFKKGKF--------------------------------------   402
p.falciparum    YIGNHQVVADLPYYELKGGRFSSTFWKVVYIQLLLSWKSRFHFFIDFIKTKWYGRPFIK   533
                               :* *:*
```

The assignment of each output alignment position follows the general scheme: * (residue identity), : (residue semi-conservation) and . (residue conservation). The alignment was carried out using ClustalO (v1.2.4) [54].

^{1}H and ^{13}C NMR spectra of the final compounds

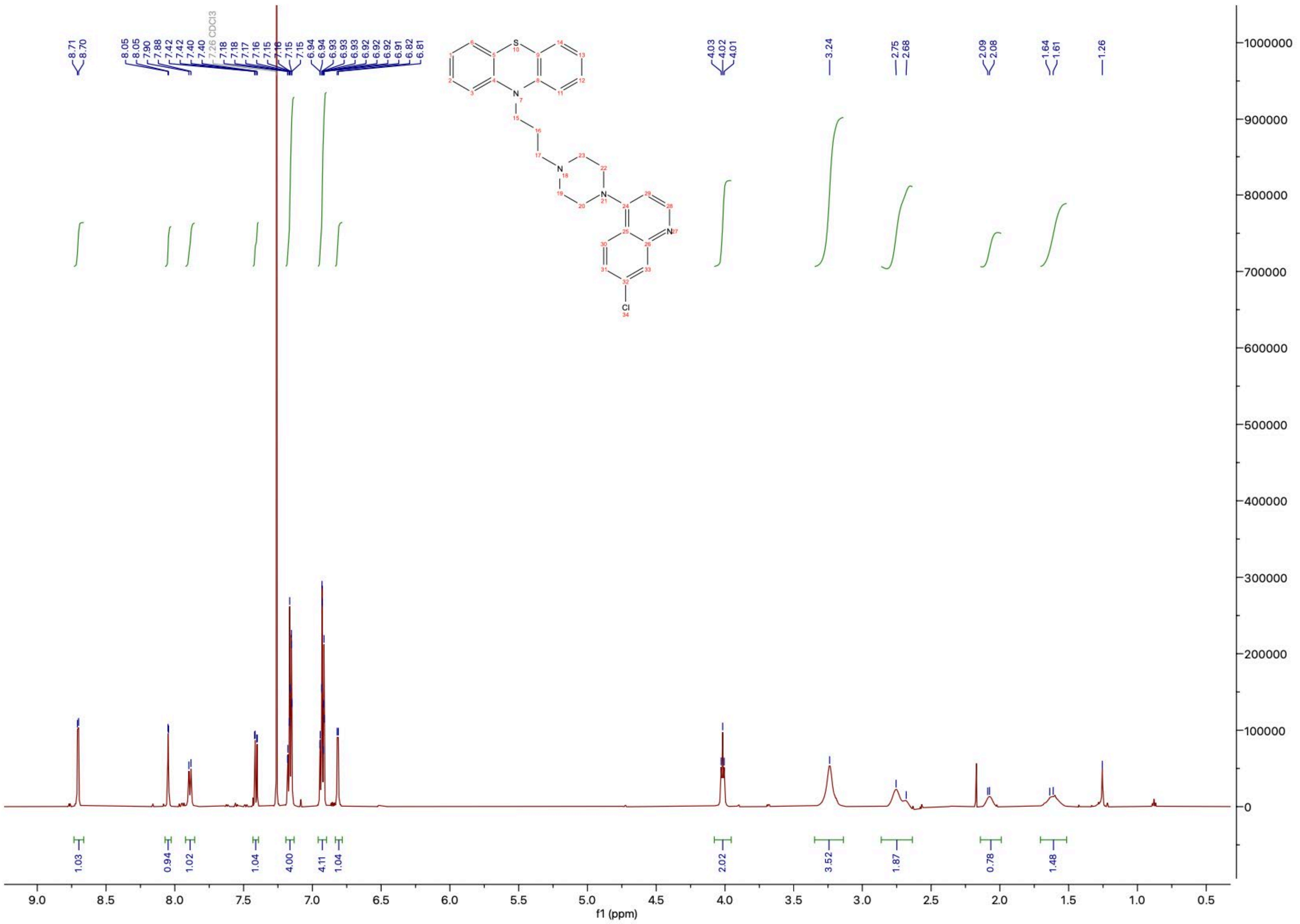

Figure S5: ^{1}H-NMR Spectra of compound **1**

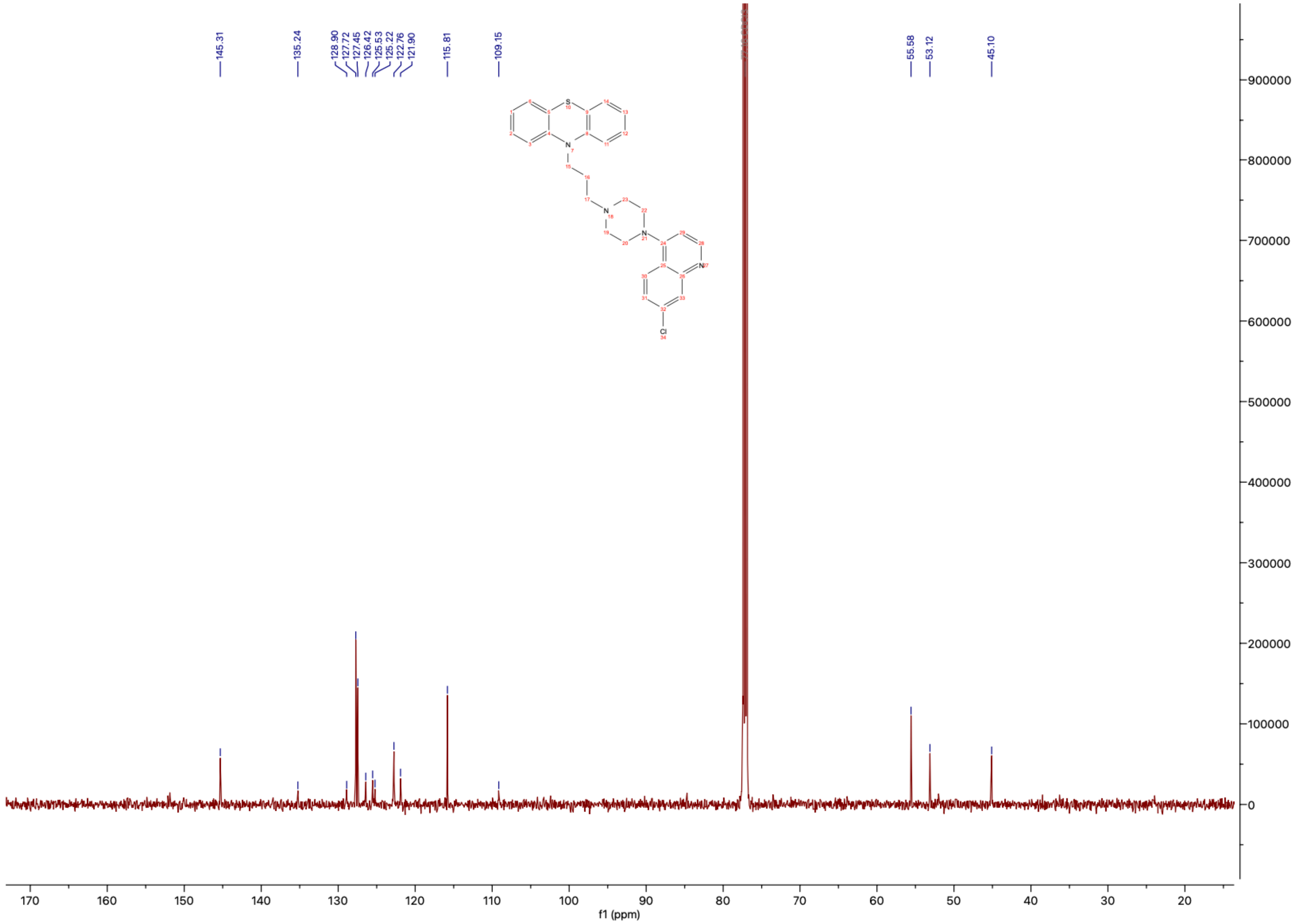

Figure S6: ^{13}C-NMR Spectra of compound **1**

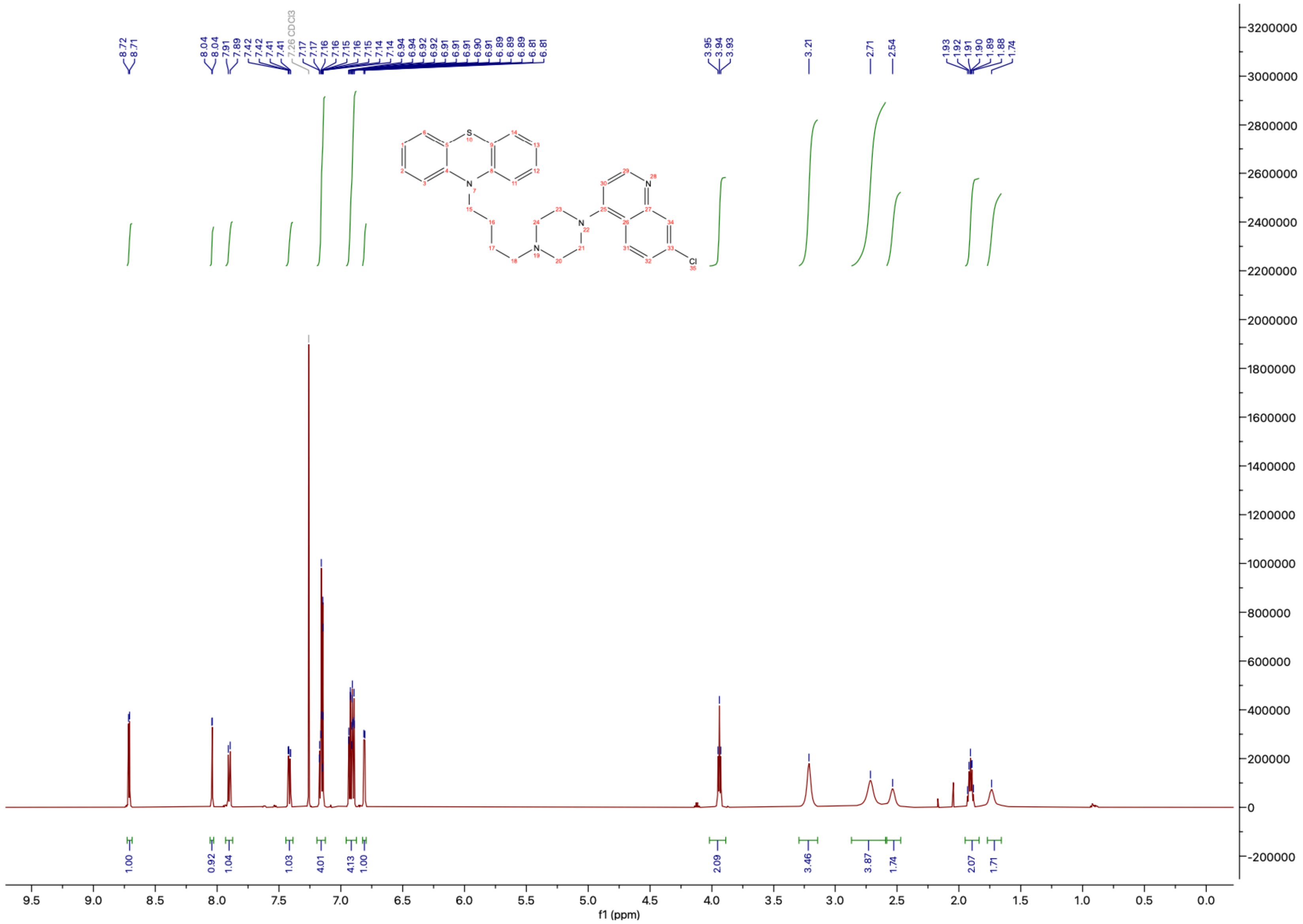

Figure S7: ^{1}H-NMR Spectra of compound **2**

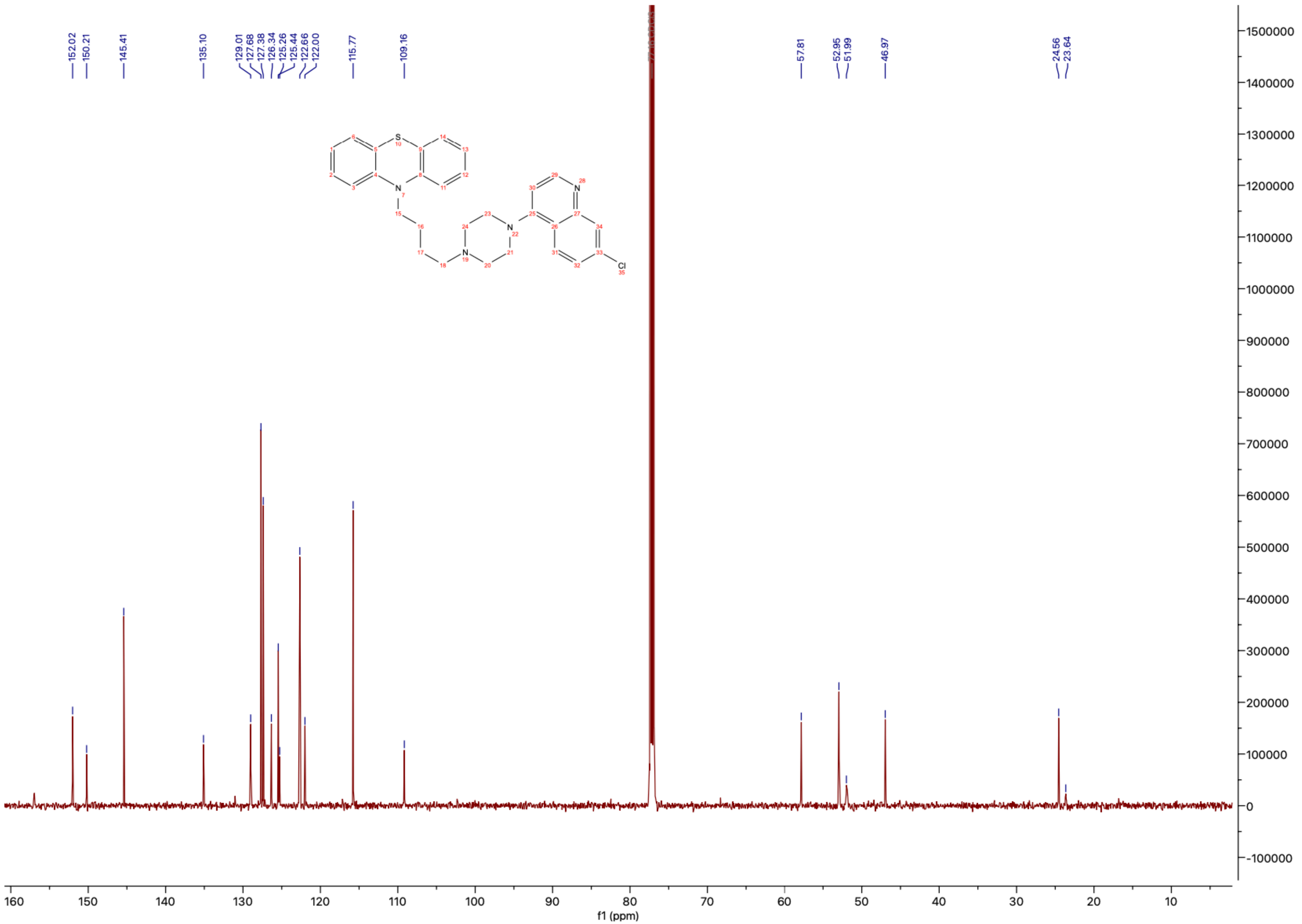

Figure S8: ^{13}C-NMR Spectra of compound **2**

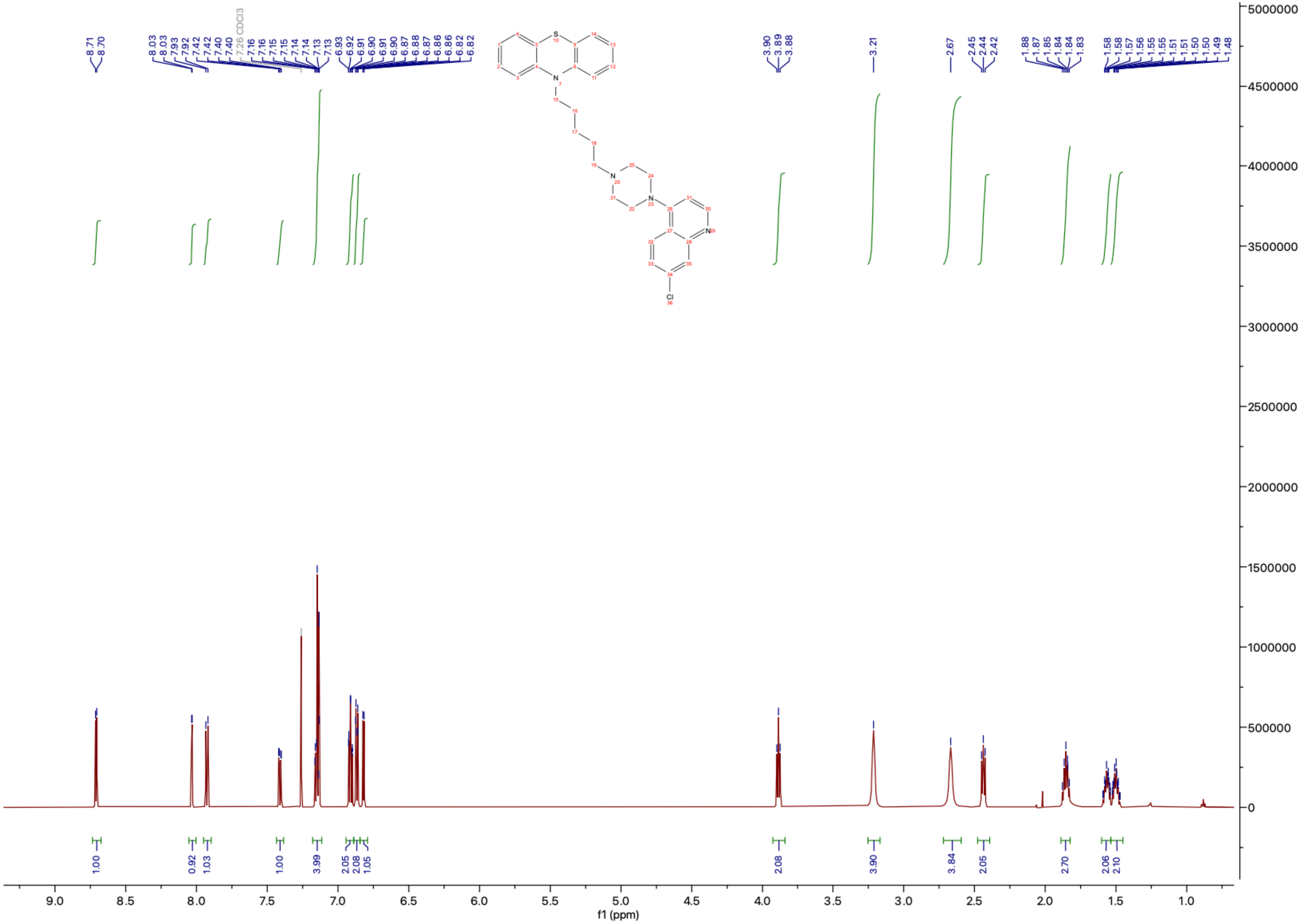

Figure S9: ^{1}H-NMR Spectra of compound **3**

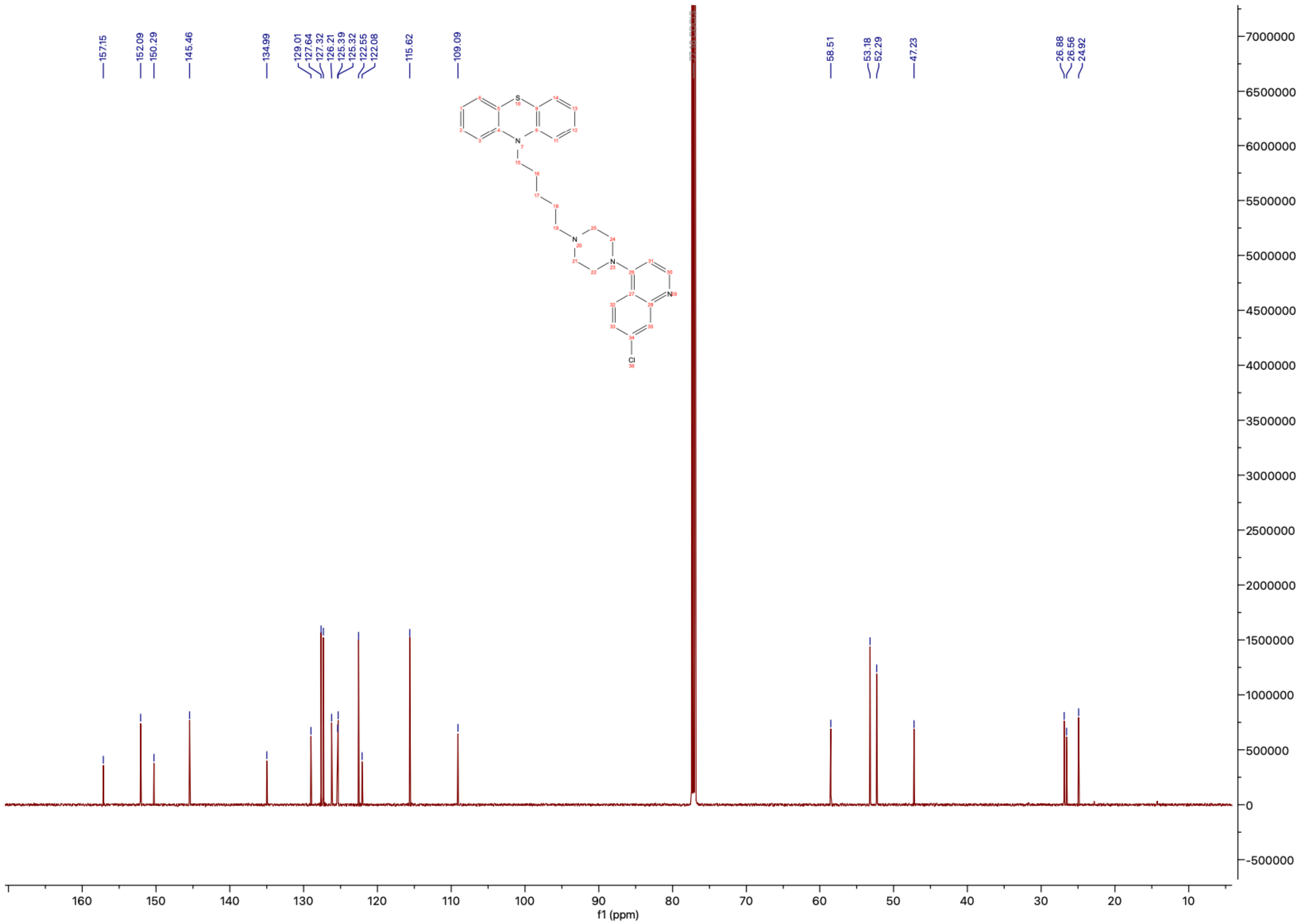

Figure S10: ^{13}C-NMR Spectra of compound **3**

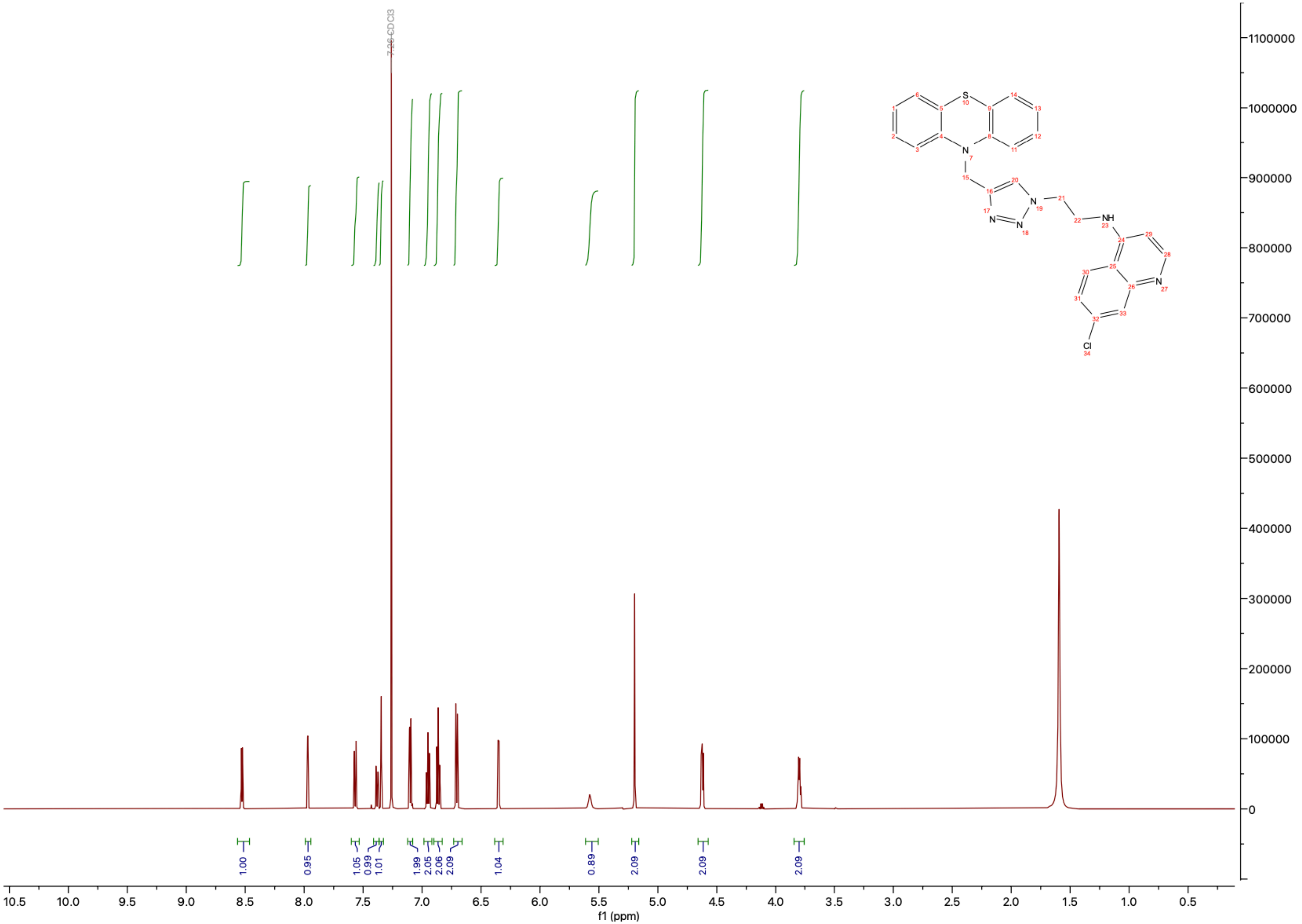

Figure S11: ^{1}H-NMR Spectra of compound **4a**

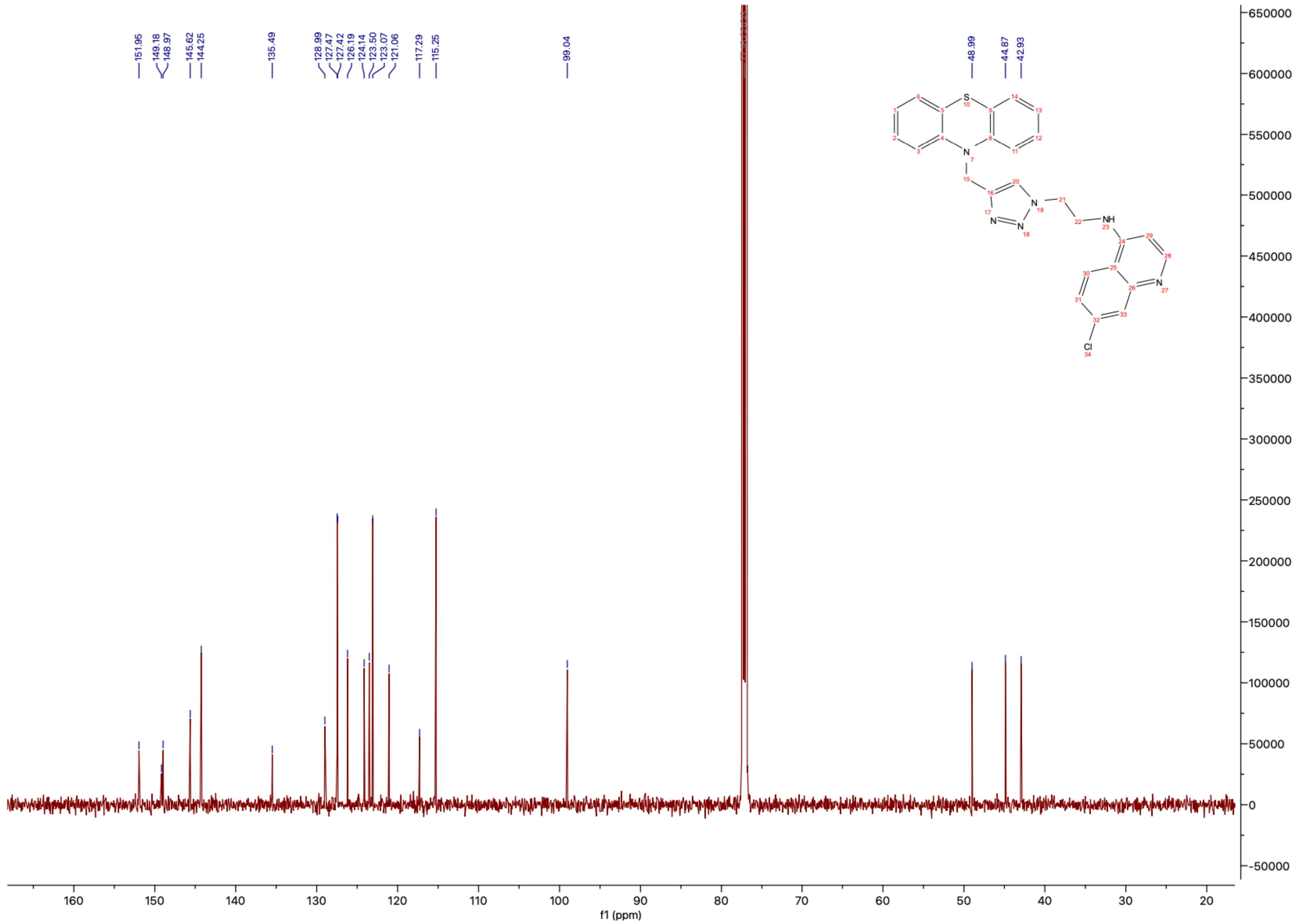

Figure S12: ^{13}C-NMR Spectra of compound **4a**

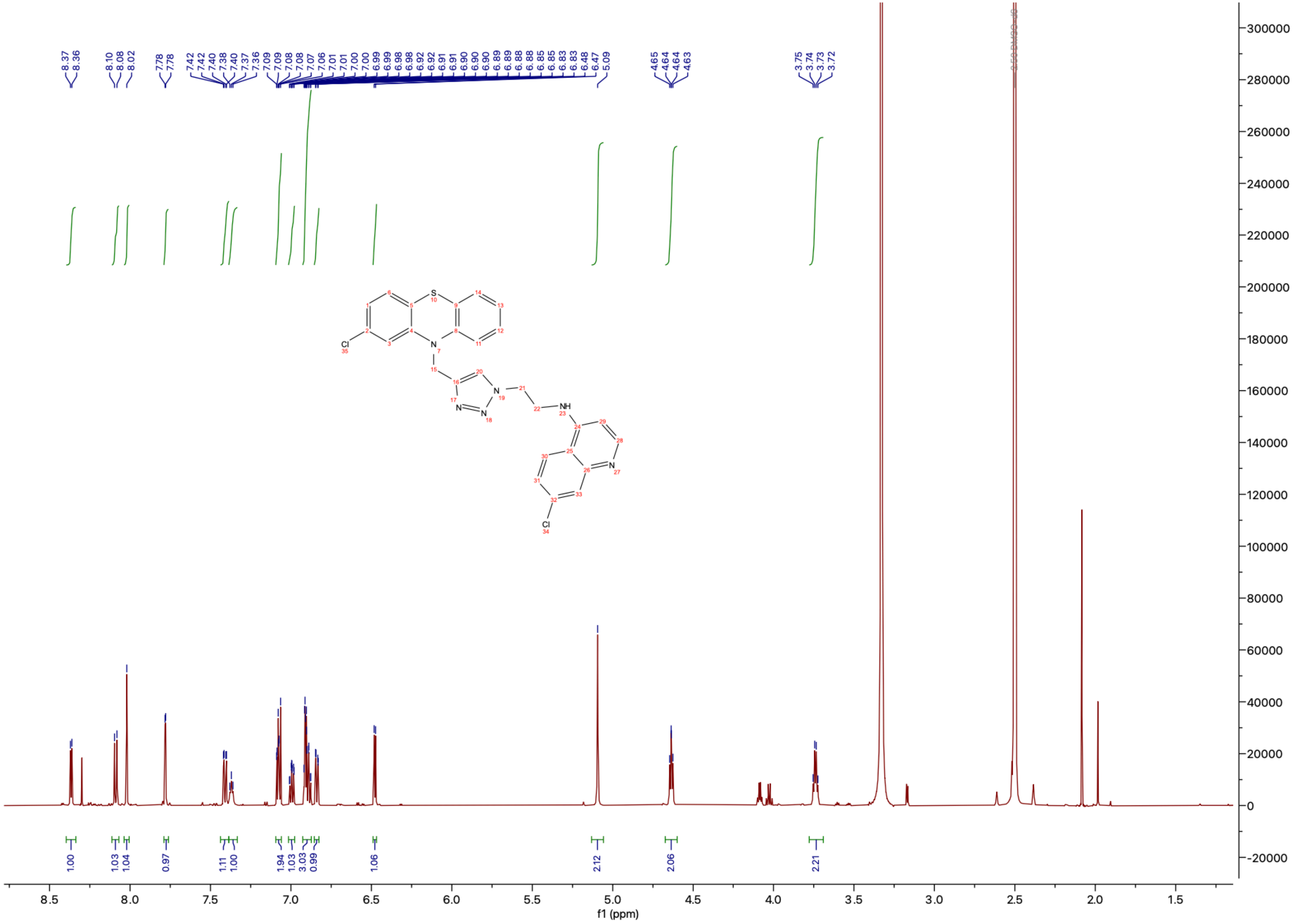

Figure S13: ^{1}H-NMR Spectra of compound **4b**

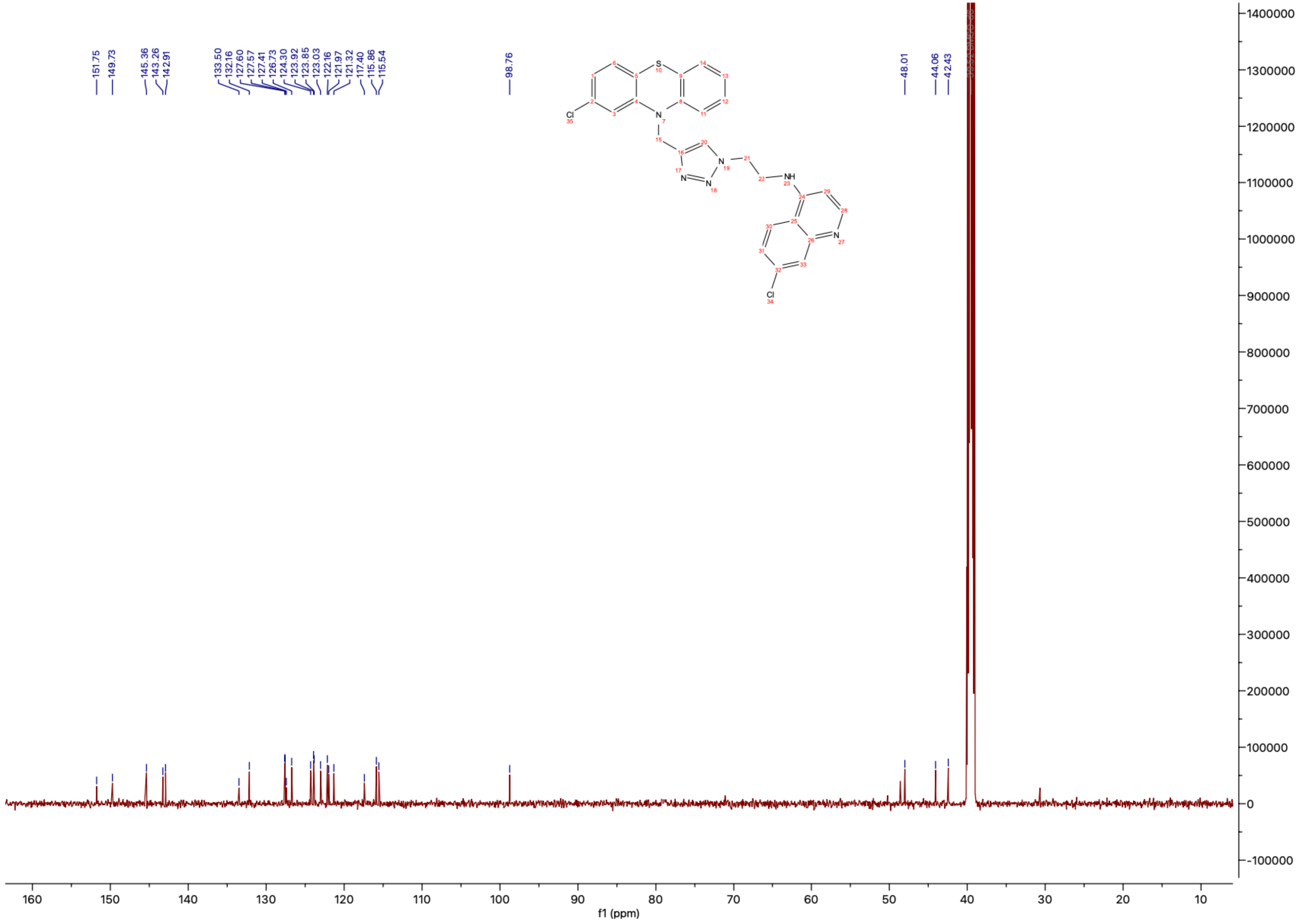

Figure S14: ^{13}C-NMR Spectra of compound **4b**

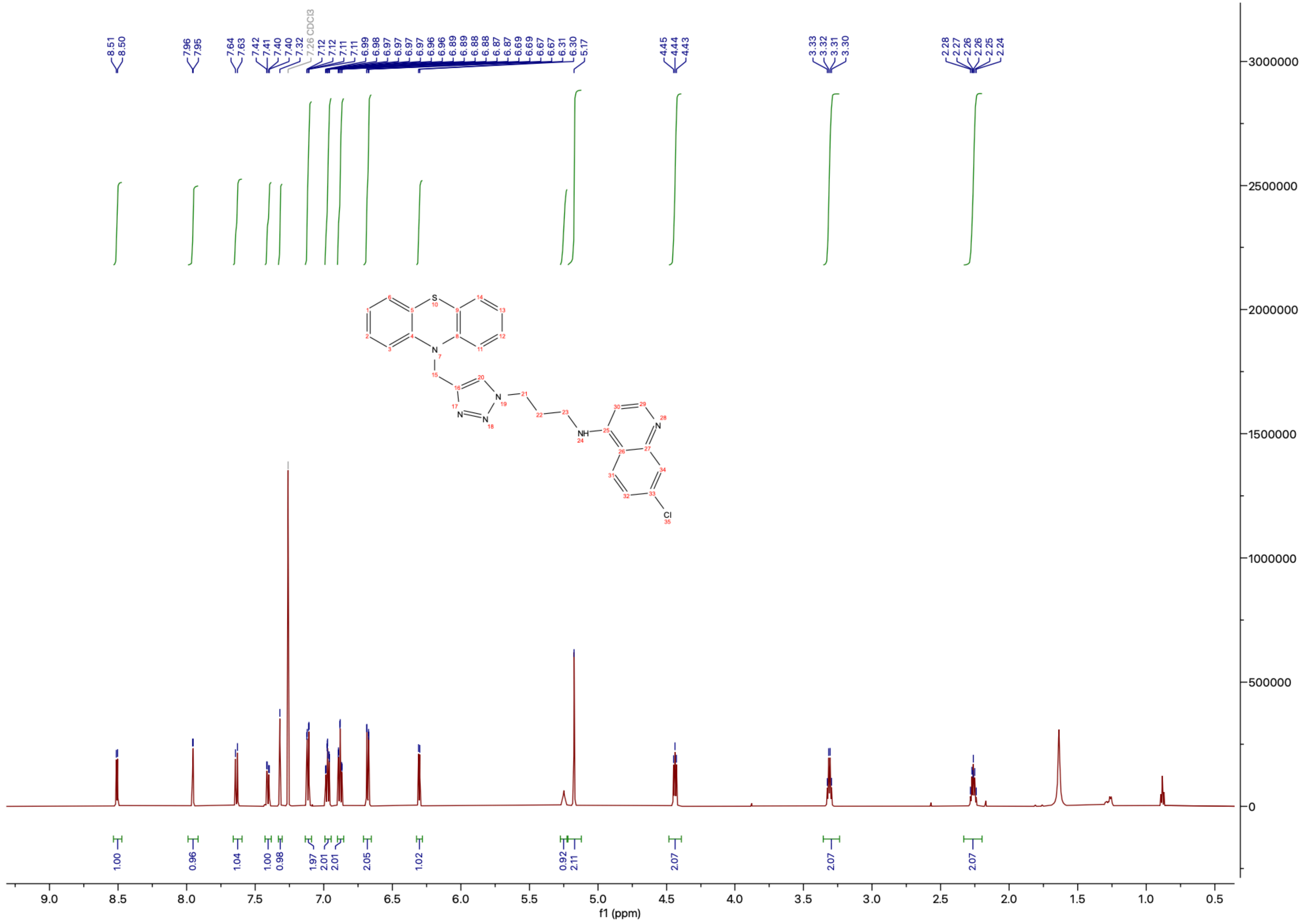

Figure S15: ^{1}H-NMR Spectra of compound **5a**

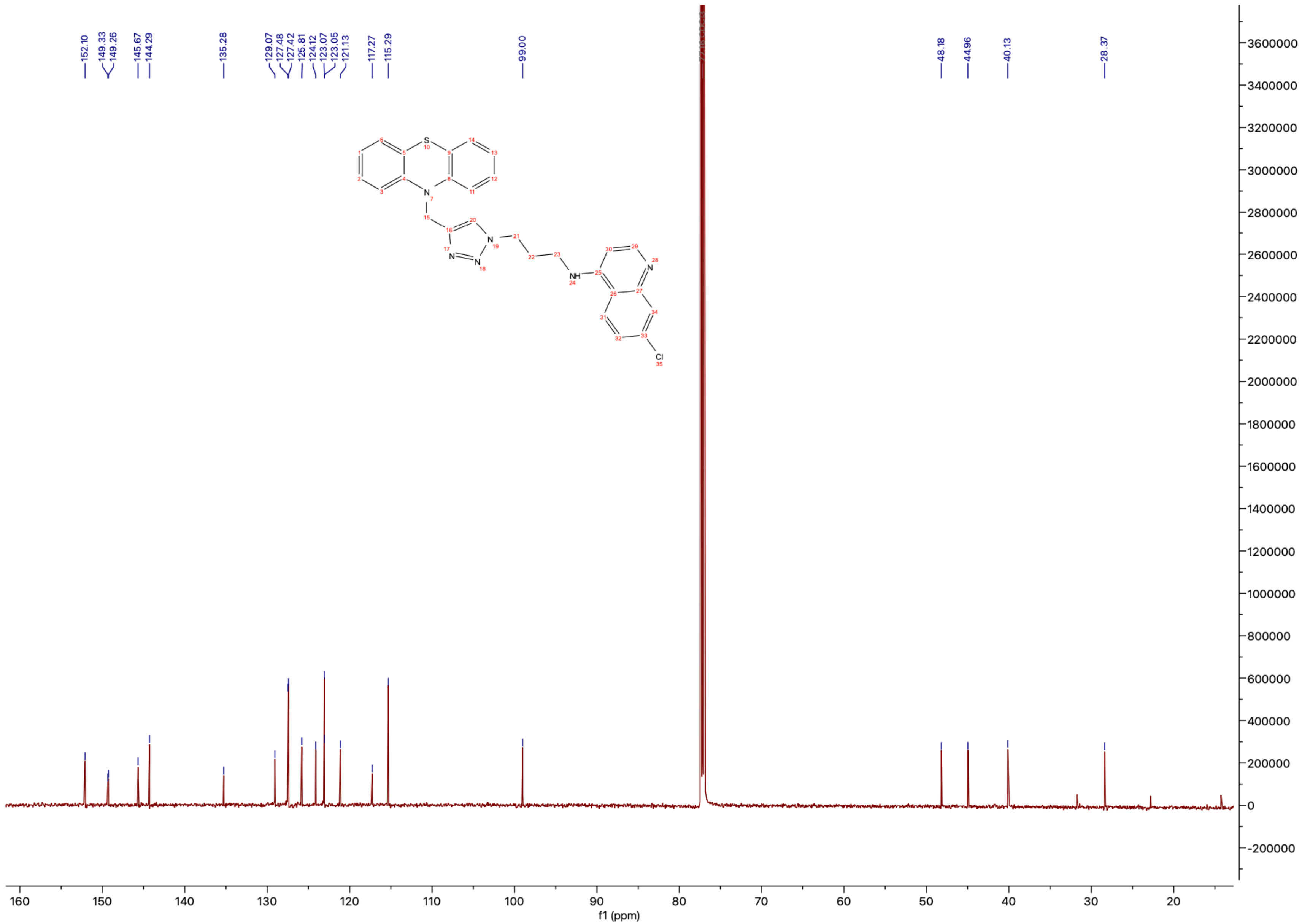

Figure S16: ^{13}C-NMR Spectra of compound **5a**

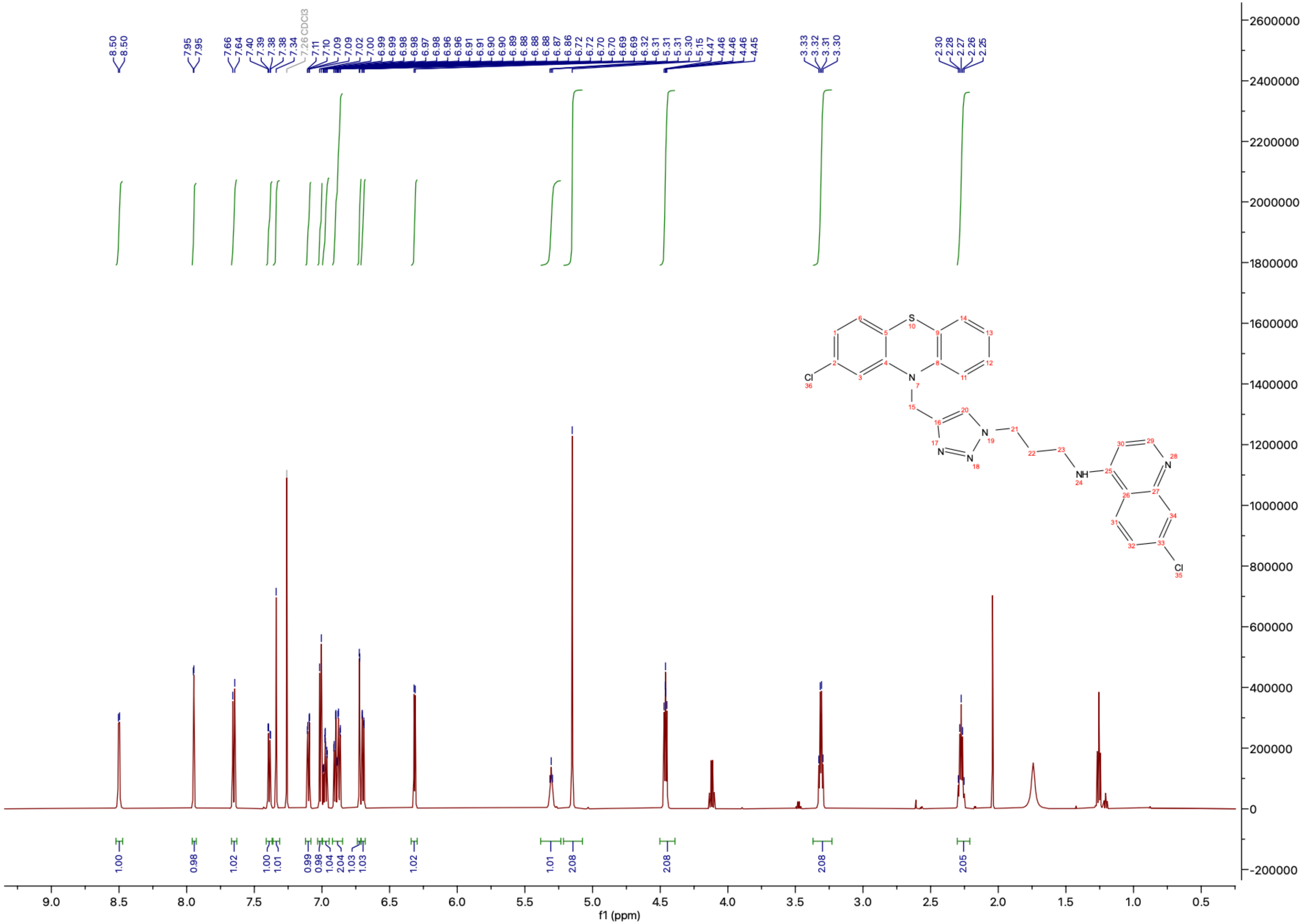

Figure S17: ^{1}H-NMR Spectra of compound **5b**

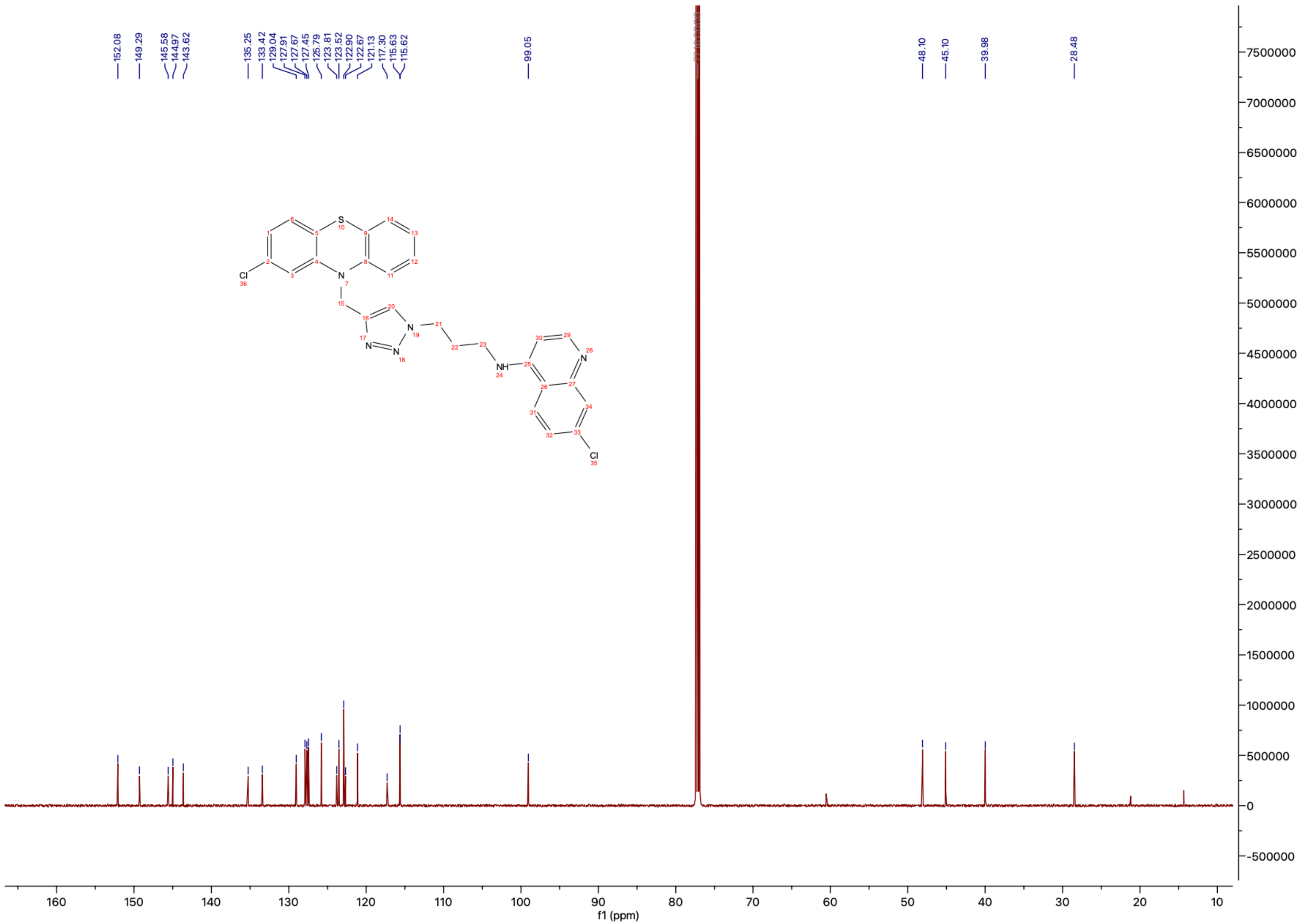

Figure S18: ^{13}C-NMR Spectra of compound **5b**

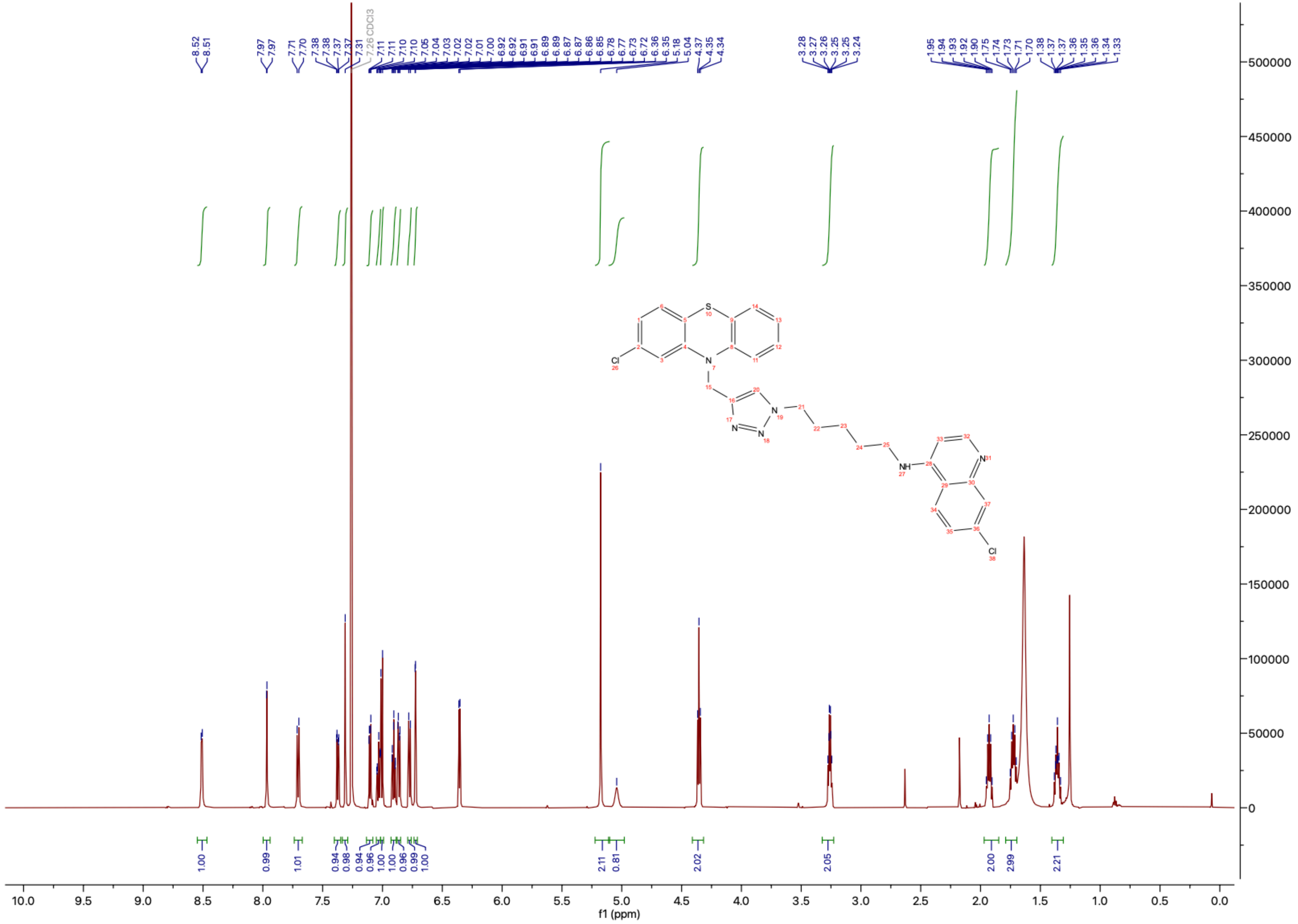

Figure S19: ^{1}H-NMR Spectra of compound **6b**

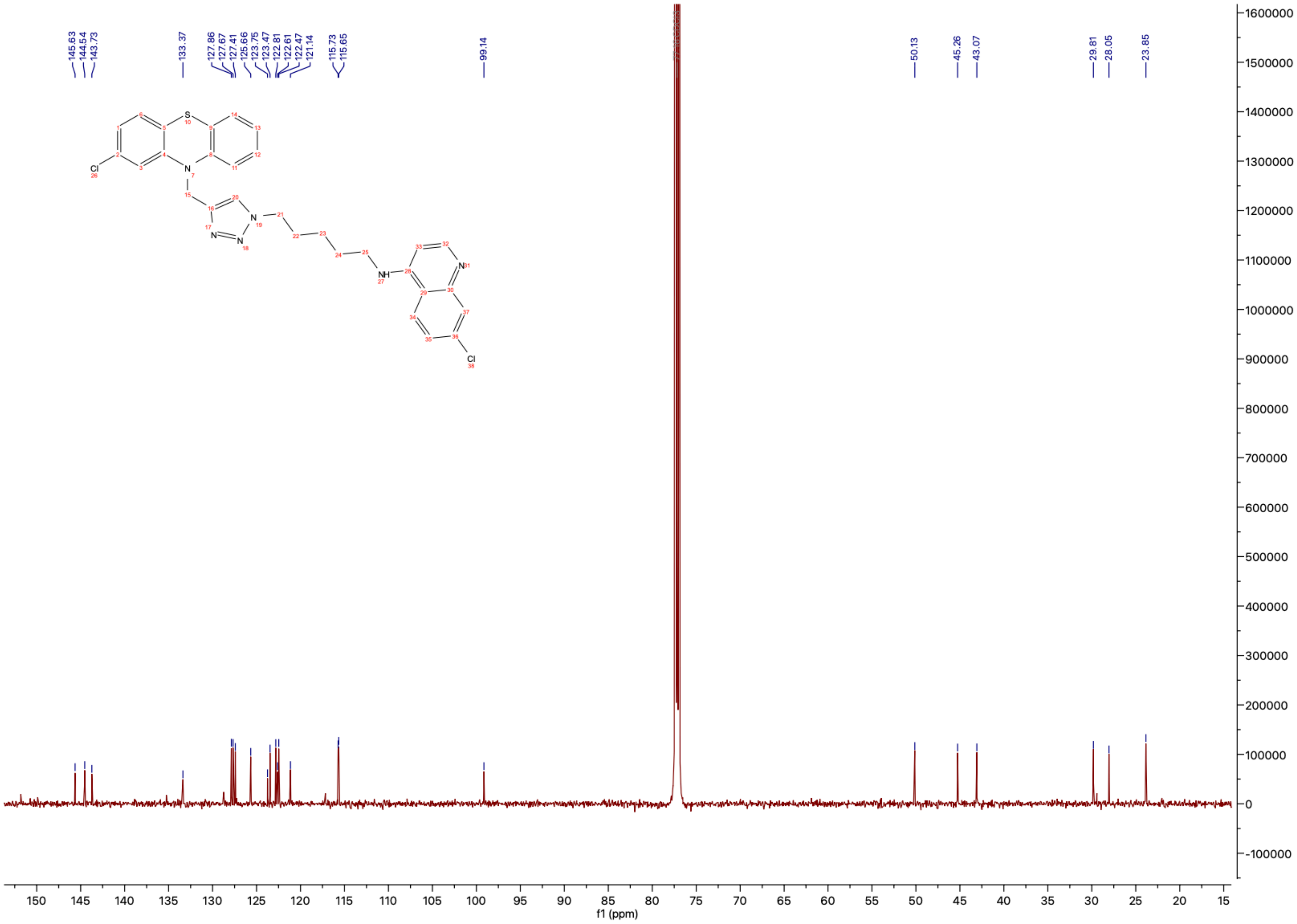

Figure S20: ^{13}C-NMR Spectra of compound **6b**

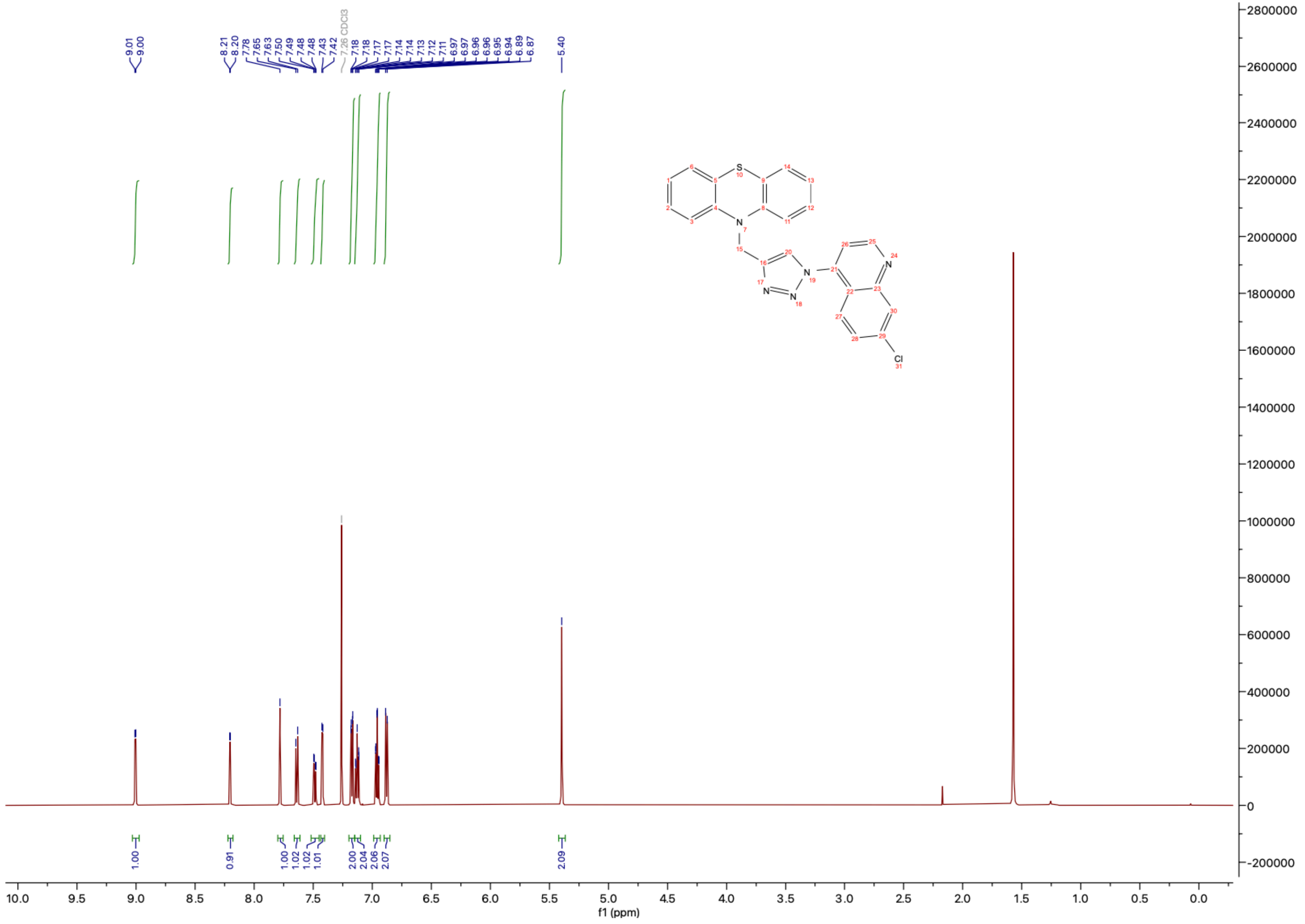

Figure S21: ^{13}C-NMR Spectra of compound **7**

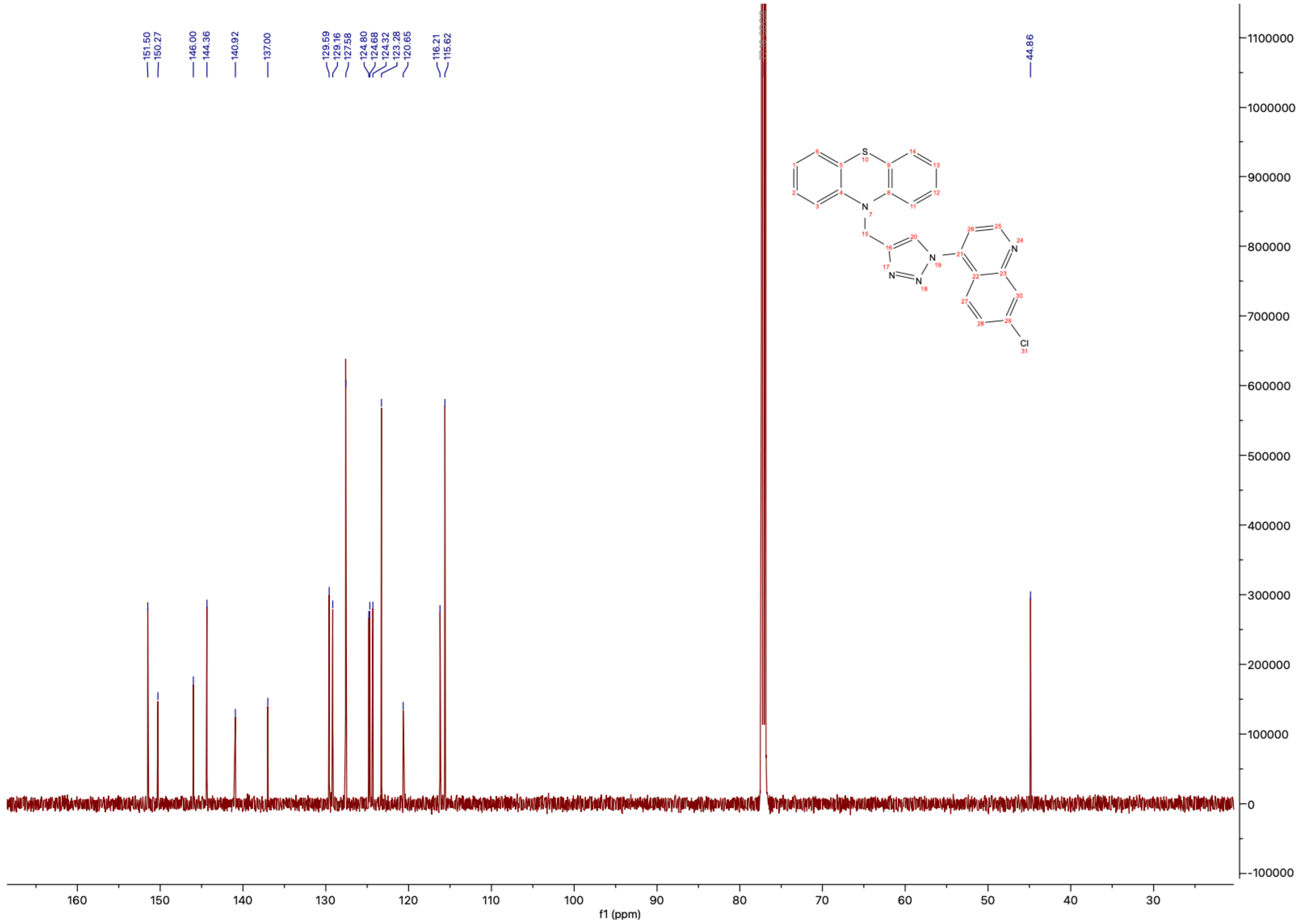

Figure S22: ^{13}C-NMR Spectra of compound **7**

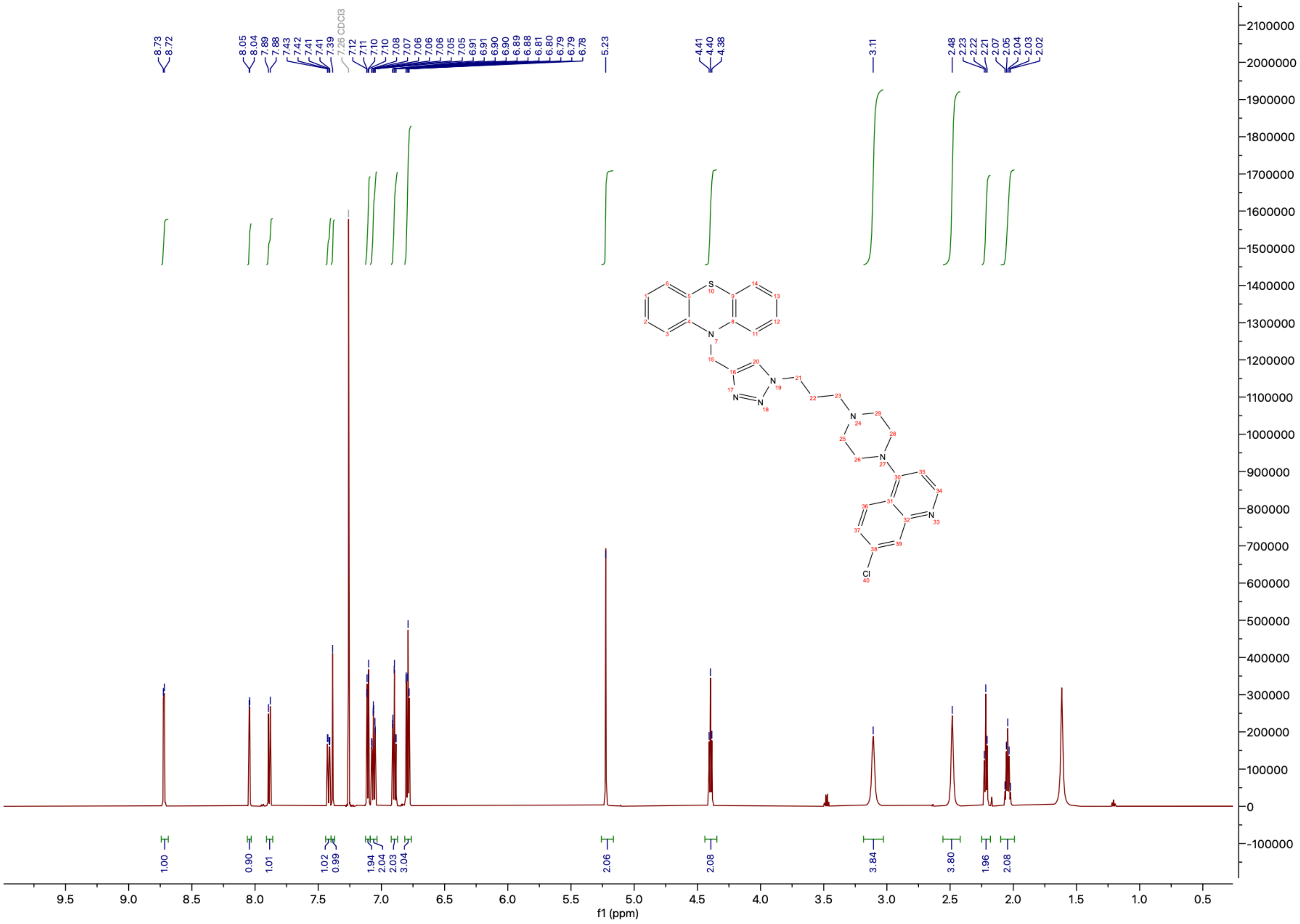

Figure S23: ^{1}H-NMR Spectra of compound **8**

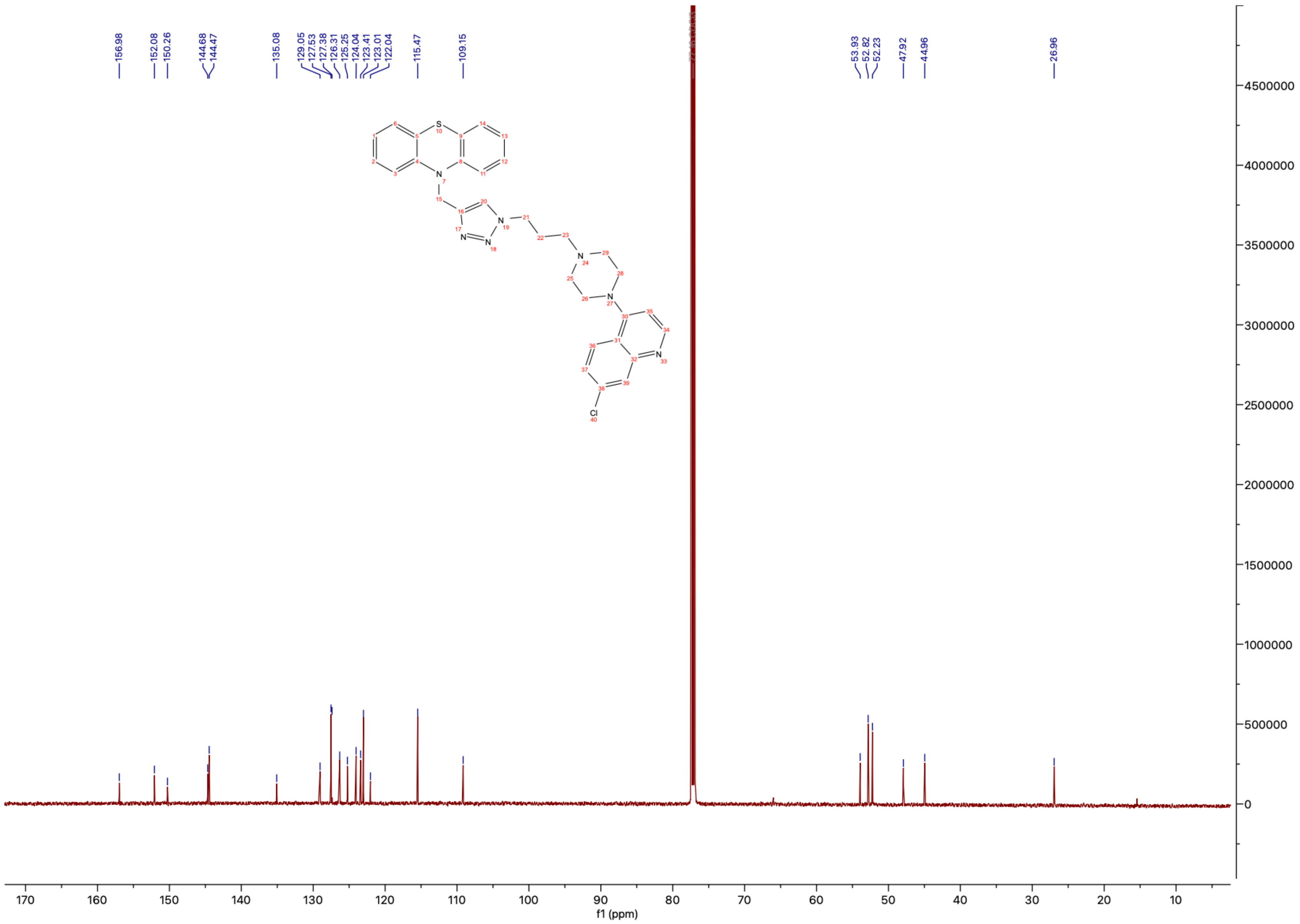

Figure S24: ^{13}C-NMR Spectra of compound **8**
